# Maternal Referral Delays and a Culture of Downstream Blaming Among Healthcare Providers: Causes and Solutions

**DOI:** 10.1093/phe/phac021

**Published:** 2022-09-21

**Authors:** Monali Mohan, Rakhi Ghoshal, Nobhojit Roy

**Affiliations:** WHO Collaborating Center for Research in Surgical Care Delivery in LMIC, Mumbai, India; CARE India Solutions for Sustainable Development, Delhi, India; Department of Global Public Health, Karolinska Institutet, SE-171 77, Stockholm, Sweden

## Abstract

Patient referral management is an integral part of clinical practice. However, in low-resource settings, referrals are often delayed. The World Health Organization categorizes three types of referral delays; delay in seeking care, in reaching care and in receiving care. Using two case studies of maternal referrals (from a low-resource state in India), this article shows how a culture of downstream blaming permeates referral practice in India. With no referral guidelines to follow, providers in higher-facilities evaluate the clinical decision-making of their peers in lower-facilities based on patient outcome, not on objective measures. The fear of punitive action for an unfavorable maternal outcome is a larger driving factor than patient safety. The article argues for the need to formulate an ecosystem where patient responsibility is shared across the health system. In conclusion, it discusses possible solutions which can bridge communication and information gap between referring facilities.

## Referred ‘Too Late’ and ‘Too Soon’: Two Case Studies From India

### Case I

22-year-old Anjum was referred by the primary healthcare center to the district level hospital for management of postpartum hemorrhage. She had unrecordable blood pressure and was clinically unresponsive when she reached the District Hospital. However, she responded to the resuscitation provided by the doctors there. They transfused her four units of blood, gave her oxytocin and sutured her cervical tears. As this was the district level health facility, certain technical support was not available at night. So, anticipating that Anjum might require higher-level care, the doctors referred her to the nearest tertiary facility which was two hours away. On the way, Anjum stayed hemodynamically stable. At the tertiary hospital, she could not receive the lifesaving surgery she needed; she suffered a cardiac arrest and died three hours later. The doctor at this apex facility called her husband in and informed him of the demise: ‘Your wife is dead’. He further added, ‘had she been brought to us sooner, we might have been able to save her’. One of Anjum’s attending doctors (coauthor MM) from the district hospital had also accompanied her to the tertiary hospital, and she noted how this doctor blamed doctors at the lower-level district facility for presumed bad decision-making. However, he had not asked her about the management they had provided at the District Hospital. She asked him what clinical interventions he would have done differently had Anjum come in sooner. When he detailed his preferred line of intervention for treating Anjum’s condition, MM explained to him that they already had done exactly those interventions at their facility, and had referred Anjum out only after that. It was after some back and forth between them that the tertiary facility doctor realized that Anjum had not really been referred ‘too late’.

### Case II

On a different day and place, at another district level hospital, coauthor RG observed a woman who was referred in from a lower facility for possible postpartum hemorrhage. There was a single doctor on duty at the female Outpatient Department (OPD) at that district hospital and this doctor was responsible for attending to the 100+ women who had come in for antenatal visits or for other gynecological issues. When the patient came in and got admitted through the emergency department, the doctor put her OPD patients on hold and went to attend to the patient. After assessment, she realized that while the postpartum bleeding was more than normal, it was yet not critical. She assessed that the lower-facility providers should have been able to manage the patient on their own and was convinced that the woman should not have referred out to the higher facility so early. She scolded the patient’s mother for having gotten her to the district hospital ‘too soon’.

## Ad hoc Referrals and the Myth of a ‘Right Time’

The above two cases took place in two districts in the state of Bihar, India. Bihar is one of the Empowered Action Group (EAG) states in eastern India with a population of 104 million which is close to the population of Philippines (Bihar Population Sex Ratio in Bihar Literacy rate data 2011-2022, n.d.). An EAG state lags in demographic transition with relatively higher fertility and mortality indicators (Arokiasamy and Gautam, 2008). The cases of maternal referrals described above work as pegs to start the conversation around referral delays and lack of coordination—two issues that are exacerbated by low resources in developing country contexts.

The responses of healthcare providers at both the higher facilities (in the above cases) implied that there is a prior known ‘right time’ for making referrals. Unfortunately, the ‘right time for referral’ is a myth. The ‘conclusion’ about whether a referral was made timely or not, is based on patient outcome, only in retrospect. Assessment of the alleged timeliness of a referral is neither protocolized nor does it depend on any prior decided indicator. Healthcare providers at higher facilities are often overburdened with patient volumes. So, on the one hand when they see that a referred-in patient who is not critical, they commonly blame healthcare providers at the referring-out lower facility for making a referral ‘too soon’. On the other hand, if the referred-in patient dies soon after reaching the higher facility, the lower-facility providers are blamed for having made the referral ‘too late’. It is the patient outcome that validates (or negates) the rightness of the referral decision made by providers at the referring-out lower facility.

The clinical or infrastructural reasons for making a referral, retrospective assessment of referral timeliness, and an impetus to downstream blame, contribute to a culture of ad hoc-ism, broken communication and lack of coordination among healthcare providers. In this article, we discuss the factors that help sustain this ad hoc culture of downstream blaming and suggest some possible ways of addressing them.

## Referral Delays: The Role of Geography and Infrastructure

Maternal health outcomes are a delicate subject. The burden is especially high in the developing world, and this was aggravated by the COVID-19 pandemic as multiple systems became partially dysfunctional. Out of all the global maternal deaths, India contributes about 15 per cent (Tripathy *et al.*, 2016). Issues including lack of resources and relatively weaker health systems contribute to the problem by obstructing timely maternal referrals. Maternal referrals play a crucial role in improving maternal health outcome (Raj *et al.*, 2015). However, most Indian states do not have a standardized referral protocol, and this leads to ad hoc and undocumented referrals, poor patient care and an atmosphere of mistrust between health providers themselves.

The World Health Organization (WHO) categorizes referral delays into three types: delay in seeking care (i.e. the delay in leaving home), delay in reaching care (i.e. the delay in transit) and delay in receiving care (i.e. the delay after arriving at the facility) (Calvello *et al.*, 2015). While delays cannot be eliminated, they can be reduced if specific barriers are addressed. The absence of standardized referral protocol in many low-resource settings adds to delays (Hamal *et al.*, 2018). A review of 1000+ maternal deaths in an EAG state in India found that pregnant or postpartum women were required to travel an average of 13 kilometers from home to reach a healthcare facility (Mohan *et al.*, n.d.). On average, the women spent five hours at the first healthcare facility before being referred out to two more facilities. One-third of all women died in transit. Hamal *et al.*’s review shows that ‘The majority of deaths in 10 Indian states (77–84 per cent) were among women who sought care—either at health facilities, during facility-to-facility referrals, while returning from a health facility or at home after returning’ (Hamal *et al.*, 2018).

A study from Haryana, India, shows that as distance between home and hospital increases, fewer women reach the hospital resulting in a decreased numbers of institutional deliveries (Prinja *et al.*, 2014). Given that states such as Haryana and Bihar are largely rural, geographical access is a major challenge and the ambulance is the primary mode of transport during an emergency. Evidence shows that when access to ambulance services increase, institutional deliveries increase, resulting in improved maternal outcomes (Prinja *et al.*, 2014). Other challenges for low-resource settings include low volumes of health providers, weak infrastructure, non-functional blood storage units lack of technical staff and laboratory services and lack of timely available transport (Singh *et al.*, 2009; Hamal *et al.*, 2018; Kironji *et al.*, 2018; Sood *et al.*, 2019). When a referred-out patient, after negotiating these barriers, reaches the higher facility, she often presents a poor outcome, which triggers the healthcare providers to blame providers in the referring-out lower facilities.

Review of maternal referrals in other low-resource settings shows a similar picture. In a Liberia county, patient referrals take place without any pre-defined referral pathways, which exposes a lack of communication between health providers, and between providers and patient/family (Kim *et al.*, 2017). With no referral protocol to refer to, lower facilities refer patients based on personal contacts they have at the next available higher facility. This breeds a clinical culture where referrals are undocumented, which in turn implies that the outcome of a referred-out patient is lost to the system as is the possibility of any follow-up (Kim *et al.*, 2017). A study from Morocco observes that within the WHO’s third delay phase, multiple referrals take place: ‘women made several trips between the health center and the peripheral maternity ward or the hospital and a university hospital’. The authors also note that even after women reached a facility, ‘they were transferred from one facility to another without receiving any information’ (Assarag *et al.*, 2015). They argue that the ‘third delay’ should be split into two sub-parts, viz. delay in reaching the first healthcare facility, and delay in reaching the final facility where appropriate management finally starts (Assarag *et al.*, 2015).

However, we argue that the authors of the Moroccan study have overlooked another layer of reality. The delays between each stage are rarely neat and discrete. Clinical management of a serious patient is not an event but a process that spreads out across the facilities the patient reaches. As Hamal *et al.* (2018) point out, within the third delay multiple referrals often take place (Hamal *et al.*, 2018). Treatment takes place in a graded manner as we saw in the two cases above—there is no one facility where treatment starts in a discrete way, since each facility plays a part in the intervention. While all the facilities that a woman visits play a supportive role in the clinical intervention, the third delay is spread over this pathway of access. A study from Nepal summed up five broad reasons for delay in referrals, especially of the third delay: (i) unavailability/limited availability of core triage and stabilization products, (ii) dearth of human resources, (iii) inadequate service delivery systems, (iv) lack of clarity of governance and finance related protocols and (v) non-robust information systems (Fleming *et al.*, 2017).

## Do These Problems Affect Health Systems Only in Low-Resource Settings?

These referral problems seem much the same for most low-resource settings such as India, Liberia, Morocco and Nepal. Solutions for addressing these challenges seem to lie in developing and implementing protocols and standard operating guidelines. Increasing resources seem to logically lead to improved functionality and coordination between healthcare providers.

These problems are not a result only of lack of resources since high-resource settings face similar issues as well. The root of these problems lies not in resources but in gaps in coordination and communication between healthcare providers. Studies show that these components are commonly lacking in high-resource settings as well. Nicholson *et al.*’s systematic review identifies a range of barriers: conflicting aspirations of different parts of the system and the need to balance the interests and values of all stakeholders involved in integrating care (Nicholson *et al.*, 2013). Lack of shared goals and a failure to regularly revisit the integration mandates, are additional barriers. Finally, the absence of documentation and evaluations of the lessons learnt add to the failures. A study on the National Health Systems in the U.K. summarizes that the barriers to effective coordination and communication include ‘a lack of commitment across organizations, limited resources, poorly functioning information technology, poor coordination of finances and care pathways, conflicting objectives and conflict within teams’ (Kozlowska *et al.*, 2018). The authors point out that education for healthcare professionals also fail to accomplish the desired coordination since education and training are often not linked to each other, and so, does not motivate healthcare professionals to work together with a shared goal.

Communication and coordination are major challenges between primary care providers and specialized care providers in high-resource settings with improved systems in place (McHugh *et al.*, 2013; Zuchowski *et al.*, 2014). With no resource constraint and with high-end technology at hand, such settings have implemented integrated service delivery platforms and use a shared electronic medical record. A study of the Veterans Health Administration in the USA show that despite fully functional electronic medical records, the health systems face similar gaps in provider coordination and communication (Zuchowski *et al.*, 2014). Interviews with primary care providers as well as with specialists showed that referrals are smoothest when the two providers know each other personally: ‘They reported using the phone or e-mail to contact specialty providers whom they knew to alert them to the needs of a particular patient and to request an appointment’. Data show that despite shared medical records and protocolized referral mechanisms, communication between providers is not necessarily smooth. The authors analyze that since primary care providers and specialists are ‘dispersed collaborators’, establishing ‘mutual knowledge is more difficult … and are prone to miscommunication’. It is worth highlighting that a lack of coordination and communication between healthcare providers are barriers that do not depend on resource availability.

## Fear of Punitive Action

In India it is mandatory to report a maternal death with 24 hours of the death. Following the information of a maternal death, an investigation takes place; the report is to be submitted within 3 weeks of reported death. Deaths which happened within a hospital are to be investigated within 24 hours. Deaths during a referral transit are included within facility based deaths as well. This Maternal Death Surveillance and Response (MDSR) is ‘a continuous cycle of identification, notification and review of maternal deaths followed by actions to improve quality of care and prevent future deaths’. (Ministry of Health and Family Welfare, Government of India, 2017) The MDSR guidelines emphasize on a pathway analysis to identify preventable referral loops and provides a comprehensive direction for reviewing each death that has happened. However, a standardized referral protocol detailing the guidelines for referral is not available.

In the absence of such a document, a major barrier is the fear of punitive action one could potentially face if the patient outcome turned unfavorable. A study of social factors contributing to maternal deaths in India found that referrals were made even by health facilities which were well equipped and did not face resource challenges (Kaur *et al.*, 2018). The likely reasons for such referrals are the provider/facility’s intention to avoid a maternal death within their own premises. A news report in 2017 foregrounded how senior healthcare providers use the MDSR mandate as a tool to threaten and insult junior providers (Tamil Nadu govt doctors to boycott maternal death audit meetings | Chennai News - Times of India, n.d.).

A scoping review of qualitative and mixed-methods studies from the public health sector in India also showed that fear of punitive actions as a limitation to report maternal deaths (Raj *et al.*, 2015; Hamal *et al.*, 2018). A qualitative study from Odisha, India, assessed the maternal death review process in the state (Naik *et al.*, 2020). It found that maternal deaths were heavily underreported owing to fear of punitive action against the healthcare providers. By extension of this fear, referrals are done in an ad hoc manner primarily to avoid potential mishap and any penalty in the future. Higher facilities have difficulty in referring out a patient, and when the patient suffers a poor outcome in their jurisdiction, they blame the lower-facility providers. However, providers at both ends of the hierarchy are part of the same fear-culture where survival of the healthcare provider assumes more importance than the treatment of the patient.

The fear of punitive action and possible legal entanglements affect providers in other settings too. An assessment of maternal death reviews in 46 sub-Saharan Africa countries found that lack of a legal framework tended to result in misconceptions and fears regarding possible punitive measures; further, the perception that maternal death audits are judgments on the actions of professional medical staff is widespread (Pearson *et al.*, 2009). A similar study in Malawi found that death audits are at times used by managers to punish the care providers (Kongnyuy and van den Broek, 2008). This practice works to nurture a culture of fear which contributes to referring patients to the next facility on an ad hoc basis.

Toward providing a solution, a district-based audit from Indonesia suggests improving communication between the district health office, the district hospital, community health center staff and village midwives to increase appropriate maternal referrals (Supratikto *et al.*, 2002). In the absence of systems enabling solidarity among healthcare providers we remain stuck with a piecemeal approach to sharing patient responsibility.

## Shared Patient Responsibility, Accountability and Integrity: Ethical Concerns

Poorly managed maternal referrals have ethical implications—not only for the patients but also for the healthcare providers. The basic principles of ethics, beneficence and non-maleficence, are compromised when referrals are made in an ad hoc manner with no accountability. As this contributes to a culture of blaming peers downstream, this compromises professional integrity as well. As discussed before, in the current scenario in most low-resource settings, patient care becomes a lower priority than avoiding punitive action for an untoward outcome.

In their paper on maternal health inequities and accountability, Hamal *et al.* (2018) study how answerability and enforceability affects maternal health inequities, including mortality. Accountability is an interdisciplinary concept, originating from domains such as political science, public administration and ethics. Their framework allows them to investigate barriers beyond health service delivery, such as governance and financing. In discussing professional accountability, they point out that ‘studies frequently highlighted issues with professional accountability—assessed against ethical standards of professionalism—of health professionals responsible for maternal deaths in terms of not discharging designated duties, showing negligence in providing health care, making inappropriate and irrational referrals, inadequate interpersonal communication, behaving in a demeaning fashion toward patients, and corruption and demanding informal payments’. Further, the review shows that ‘during referrals, health professionals often did not stabilize women before referring them contributing to deaths enroute or soon after arriving at the referred facility’. A lack of accountability leads to violation of ethical principles and inhibits solidarity between healthcare providers. We believe that solidarity, as an ethical principle, is a core requirement in developing a culture where a patient is a shared responsibility between health care providers.

A patient is the responsibility of the health system, not of individual health care providers; health care providers are agents of the system who deliver the care. The ideal conditions for building a culture of shared patient responsibility is a system where the members strive to conceptualize accountability as a collaborative and cooperative process as opposed to a punitive process imposed by outside forces. This approach can be viewed as moving from a vertical to a horizontal structure or from a ‘top-down’ to a ‘roundtable’ approach as Durch *et al.* propose (Durch *et al.*, 1997). Patient responsibility needs to become a health system property rather than an individual responsibility. Ultimately, all stakeholders, together, are responsible to see that no harm occurs to the patients. In the specific domain of nursing care, Ballard *et al.* explains what is needed is a workplace environment is that it successfully supports the delivery of nursing care to the satisfaction of both the nurses and the patients (Patient Safety: A Shared Responsibility, n.d.). Ham *et al.* talks about the National Health Service and notes that imbalance of power between patients and staff, and between people and the public agencies who serve them, helps explain why change has been slow toward a shared decision-making (Shared responsibility for health | The King’s Fund, n.d.). In a similar manner, there is hierarchical imbalance between different cadres of a health pyramid where the workers with lesser health care resources are usually blamed for poor patient outcomes. The proposition of a change from professional roles to cooperation among the clinicians within a health system is one that is long overdue, and this would be the scenario in which the shared responsibility model would be able to flourish.

## Whose Responsibility Is It To Implement Safer Protocols?

The greatest disincentive to safety event reporting cited in the literature is a punitive approach to safety and the associated fear of retribution (Siewert *et al.*, 2019). An understanding that all healthcare workers are humans, make mistakes and need support, requires a culture shift which is foremost the responsibility of the organizational leadership (health facility administrators and managers).

The responsibility for implementing the protocols lie with all stakeholders within the system. Policy makers need to emphasize the need for having a standard protocol across a setting. It is the responsibility of organizational leadership to ensure that each maternal referral case is handled as per protocol. A periodic internal audit would help assess the uptake and take corrective actions for the future. An audit to understand the reason for lapses, without punishing the treating doctor will eventually dispel apprehensions about the protocol being a detriment for health care providers. Healthcare workers involved in an adverse patient event are akin to ‘second victims’, after the patient themselves, and need to be supported as such (Quillivan *et al.*, 2016). Finally, for the healthcare providers themselves, actively following the protocol—at the lower-facility end and at the higher-facility end—would be part of their commitment to the process.

## Possible Solutions for Improving Referral Quality Beyond ‘More Resources’

Using two case studies from a typically low-resource setting, this article has established the links between mismanaged referrals and a systemic culture of blame and mistrust among healthcare providers. It has shown that only a part of the barriers is because of lack of resources because a major barrier, viz. fear of punitive action, is not dependent on resources. Barriers in high-resource settings reveal the existence of gaps that stem from a lack of coordination and communication. In a punitive culture individuals are held responsible for all errors, including system errors whereas a just culture is based on the understanding that errors occur in health care and that competent professionals make unintentional mistakes (Siewert *et al.*, 2019). Taking these learnings into account, the article identifies two major steps in improving referrals and the culture of health care providers sharing patient responsibility.

Much of the literature from low-resource settings have shown that the absence of a protocol is a primary reason for the chaos in the referrals. The first step is, thus, to develop and implement across states a standardized protocol for delineating indicators and conditions for referrals potentially incorporating the uncertainty that is inherent to referrals. India has a Reproductive and Child Health referral guidelines that recommend healthcare providers at the lower referring out facilities to prepare a referral note and intimate the higher facility about the referral (Establishing a Referral Mechanism to Deliver Reproductive, Maternal, Newborn & Child Health Services | The Challenge Initiative, n.d.). However, this is only a recommendation, and its uptake has been unnoticeable. Only a few states such as Tamil Nadu (C. Vijayabaskar, Minister for Health, Tamil Nadu, 2018), Uttar Pradesh (Department of Medical, Health and Family Welfare, Lucknow, n.d.) and Madhya Pradesh have referrals management guidelines (C. Vijayabaskar, Minister for Health, Tamil Nadu, 2018; Department of Medical, Health and Family Welfare, Lucknow, n.d.; Referral Transport Ambulance System in India | UNICEF Office of Innovation, n.d.).

The second step is to focus on improving communication and coordination between health care providers across the tiers; by extension, this includes not passing down blame and adopting a non-punitive approach in case of an unfavorable outcome. The referral guidelines used by the state of Uttar Pradesh in India stresses on clear communication between the two facilities regarding the clinical condition of the patient, documenting the reason for referral and providing details of diagnosis and investigations. It further states that, ‘Even if the doctors in the receiving institution feel that the patient should have been managed in a different way, no open comments should be made at the referring end which can undo the morale of either party in the presence of the patients or relatives or over phone to any person in this regard’. In Case I, the tertiary level doctor commented on the supposedly poor decision-making by the lower-facility health care provider. Reacting to this allegation, the following day, a mob manhandled the doctors at the Primary Health Center which was the first facility that the woman had accessed. The mob locked up and manhandled the ambulance driver for alleged delay in driving the ambulance. Unfortunately, this is not an uncommon outcome for such scenarios in India.

Siewert *et al.* document three-tiered countermeasures to eliminate human factor barriers in reporting safety events by healthcare workers, at—(i) organizational/department leadership level, (ii) leadership employee level and (iii) frontline employee level (Siewert *et al.*, 2019). Building on these measures, we propose a set of possible solutions in our settings. At the organizational level, the leaders can recognize that the intervention and referral efforts made by the lower-facility health care providers were well-intentioned and geared to stabilize the patient even they are retrospectively adjudged as ‘delayed’ or ‘too soon’. A referral concern escalation program to effectively cater to the patients and health care workers could help manage expectations and hence generate a better acceptance toward the outcomes. The healthcare providers at the higher facilities (leadership employee level) can help provide positive reinforcement to the ones at lower facilities for making the referral that they could not have treated adequately. The healthcare workers at the lower facilities (front line employee level), can make the patient and the family aware of the uncertainties in the complex medical environment before referring out the patient.

In addition, if lower-health care facilities send usefully detailed referral notes addressed for the providers at the next higher facility when referring out a patient, it would initiate a culture of sharing patient responsibility. Health care providers at the next higher facility would be able to respond better to the case, and also, have an better idea of why the patient was referred. Despite an unfavorable patient outcome, practices of direct coordination and effective communication are needed. This, coupled with emphasizing non-punitive action and a positive approach in understanding referral delays shall contribute to fostering a systemic culture of trust and dependability among healthcare providers.
